# Sleep and geriatric syndromes in elderly emergency patients in China: a cross-sectional survey

**DOI:** 10.7717/peerj.20194

**Published:** 2025-10-14

**Authors:** Qiong Zhang, Peng Li, Lingyun Lu, Cheng He, Dan Chen, Fen Xiang, Can Huang, Xingliang Gan, Feng Yi

**Affiliations:** 1 Department of Emergency Medicine, Yueyang Central Hospital, Yueyang, China; 2 Department of Neurosurgery, Yueyang Central Hospital, Yueyang, China; 3 Tongji Medical College of Huazhong University of Science and Technology, Wuhan, China; 4 Department of Nursing, Yueyang Central Hospital, Yueyang, China

**Keywords:** Sleep, Geriatric syndromes, Elderly, Emergency patients

## Abstract

This study aims to assess the prevalence of abnormal sleep conditions and geriatric syndromes in elderly emergency patients in China and to explore the relationship between them. The convenience sampling method was used to recruit elderly patients in the Emergency Department of Yueyang Central Hospital in Hunan Province from July to November 2023. A total of 205 elderly emergency patients were included. Sleep conditions and four geriatric syndromes (frailty, sarcopenia, malnutrition, and cognitive impairment) were investigated. Logistic regression analysis was used to assess the relationship between sleep and the four geriatric syndromes. After adjusting for age, gender, marital status, education level, and number of comorbidities, it was found that patients with mild and significant daytime sleepiness were more likely to have frailty than those with no daytime sleepiness (OR = 2.509, *p* = 0.018; OR = 4.395, *p* = 0.048). Patients with mild and significant dissatisfaction with sleep quality were more likely to have sarcopenia than those with good sleep quality (OR = 4.153, *p* = 0.006; OR = 5.955, *p* = 0.013). Additionally, patients with normal sleep duration had a lower risk of malnutrition than those with insufficient sleep duration (OR = 0.353, *p* = 0.043), and patients with slight daytime sleepiness had a higher risk of malnutrition than those with no daytime sleepiness (OR = 3.414, *p* = 0.004). Finally, patients with mild daytime sleepiness were more likely to have cognitive impairment than those without daytime sleepiness (OR = 2.564, *p* = 0.026). This study indicates that improving sleep may be a favorable factor for controlling geriatric syndromes. However, as the single-center design and convenience sampling restrict generalizability, the results should be validated in multi-center studies using probability sampling.

## Introduction

China is home to the world’s largest elderly population. By 2023, individuals aged 65 and above are projected to constitute approximately 15.4% of China’s total population (National Bureau of Statistics of China, 2024), and researchers anticipate that the population aged 60 and above will surpass 400 million by 2035, representing over 30% of the total population (National Health Commission, 2022). The steady increase in the elderly population has introduced new challenges to the healthcare sector, particularly in the emergency medical system. Studies indicate that patients aged 65 and older account for 38% of emergency medical services in the United States, nearly four times the percentage of younger patients. In Europe, the number of emergency patients is nearly equivalent to one-quarter of these countries’ populations. However, critically ill patients aged 60 and above account for over 60% of all patients in China (Emergency Medicine Branch of China International Exchange and Promotion Association for Medical and Healthcare, Emergency Medicine Branch of Chinese Medical Association & Basic and Translational Medicine Branch of Chinese Geriatrics Society, 2024). Older patients often encounter more complex health issues with atypical symptom presentation, leading to greater diagnostic and therapeutic difficulties compared to younger patients. Due to the decline in various organ functions and the instability of the internal environment, elderly patients are prone to recurrent acute episodes and rapid disease progression. Consequently, elderly patients admitted to the emergency department tend to have poorer prognoses and higher hospitalization rates, return visit rates, and mortality rates (Aguirre et al., 2024).

Geriatric syndrome (GS) is a prevalent condition among older adults, characterized by a set of clinical manifestations that result from multiple diseases or factors (Li et al., 2023). According to Wang et al. (2024), 90.5% of older adults experience at least one geriatric syndrome, and 72.8% suffer from multiple syndromes. Among elderly patients presenting to the emergency department, 76.5% are frail, 64.8% have nutritional deficiencies, 48.4% exhibit cognitive impairments, and 64.5% require polypharmacy (Lin et al., 2021). Geriatric syndromes not only exhibit high prevalence but also significantly impact patient prognosis. For example, Carretero et al. (2023) found that malnutrition upon admission is associated with poor short-term and long-term outcomes in elderly patients. Moreover, frailty, sarcopenia, and cognitive impairment have been shown to be strongly linked to adverse health outcomes, including reduced survival rates, increased postoperative complications, prolonged hospital stays, higher incidence of falls and fractures, metabolic disturbances, cognitive decline, and elevated mortality (Yuan & Larsson, 2023; Yang et al., 2023; Fhon et al., 2023). Consequently, the traditional emergency care model is increasingly inadequate for addressing the complex needs of elderly patients, highlighting the urgent need for more specialized and comprehensive approaches to care.

Sleep plays a vital role in maintaining health (Baranwal, Yu & Siegel, 2023). Insufficient sleep has adverse effects on various physical functions, including energy metabolism, mood, and cognition (Kroeger & Vetrivelan, 2023). Sleep disorders are prevalent among elderly patients. Elderly individuals typically exhibit phase-advanced melatonin rhythms and reduced slow-wave sleep, contributing to perceived poor sleep quality (Gulia & Kumar, 2018). Mannion, Molloy & O’Caoimh (2019) found that 72% of elderly emergency department patients reported impaired sleep quality, and 13% had clinical insomnia. Sleep structure and continuity in elderly individuals change significantly and are often characterized by decreased slow wave sleep (SWS), rapid eye movement (REM) sleep, total sleep time (TST), sleep efficiency, and increased sleep onset latency (Moraes et al., 2014). The internal sleep-wake cycle is also typically phase-advanced in older adults, leading to earlier bedtimes and wake-up times (Li, Vitiello & Gooneratne, 2022). These changes are not solely due to aging but are influenced by both intrinsic and extrinsic factors (McCarthy, 2021). Evidence suggests that alterations in sleep architecture exacerbate cognitive decline and other geriatric diseases (Taillard et al., 2021; Fan et al., 2022). Sleep disorders may therefore not only be a consequence of geriatric syndromes but also a potential contributing factor to their development (Shibuki et al., 2023; Wong & Lovier, 2023). Therefore, early identification and management of sleep disorders may play a crucial role in reducing the risk of geriatric syndromes and improving quality of life.

Elderly emergency patients represent a key but neglected population in current research on sleep and geriatric syndromes. Therefore, this study focuses directly on Chinese elderly emergency patients in order to evaluate their sleep patterns and the prevalence of geriatric syndromes specifically within the Chinese emergency setting and to explore the relationship between sleep quality and various geriatric syndromes. This study will hopefully encourage medical staff to pay greater attention to the sleep status and geriatric syndromes of elderly emergency patients, thereby improving the quality of medical care for this vulnerable group.

## Materials and Methods

### Study setting and participants

Elderly patients were recruited from the emergency department of Yueyang Central Hospital in Hunan Province from July to November 2023. As the sample is based on convenience sampling, the results may not be fully representative of all elderly ED patients in China. The sole inclusion criterion was an age ≥ 60 years, and the exclusion criteria were severe physical conditions that posed an immediate threat to life and required emergency intervention. Information regarding the research procedures, objectives, related risks, and potential benefits was provided to all eligible patients. They were then invited to participate and provided written informed consent. The participants then underwent face-to-face evaluations conducted by professionals trained in comprehensive geriatric assessment. The study protocol was approved by the Ethics Committee of Yueyang Central Hospital (Approval Number: 2023-030). All research and methods were performed in accordance with relevant guidelines and regulations. According to Kendall’s (2004) method for rough sample size estimation, the sample size should be at least five to 10 times the number of variables. In this study, with 15 variables, the recommended sample size would range from 75 to 150. A total of 205 elderly patients were ultimately included.

### Sleep assessment

We used four questions to assess patient sleep status. Sleep duration was evaluated using the question: “How many hours do you sleep each night?” Participants were categorized into three groups based on their self-reported sleep duration: insufficient sleep (≤ 6 h), normal sleep (6–8 h), and excessive sleep (≥ 9 h). Daytime sleepiness was assessed using the question: “How do you think you have experienced daytime sleepiness in the past month?” Sleep quality was evaluated by asking: “How do you feel about your sleep quality in the past month?” Participants were instructed to carefully reflect on their sleep experiences over the past month and provide self-assessments of both daytime sleepiness and sleep quality. If symptoms occurred at least three times per week, participants circled the corresponding response. To assess the use of sleeping pills, participants were asked: “How often have you taken sleeping pills in the past month?” Based on their self-reported responses, participants were categorized into one of three groups: no use, occasional use, and long-term use.

### Geriatric syndrome assessment

The Rapid Geriatric Assessment (RGA) is widely used to evaluate geriatric syndromes in elderly emergency patients. This scale was developed by Morley & Adams (2015) based on the Comprehensive Geriatric Assessment (CGA) and comprises four subscales: FRAIL, SARC-F, Simplified Nutritional Appetite Questionnaire (SNAQ), and Rapid Cognitive Screen (RCS), totaling 18 items. It primarily assesses core health issues in elderly patients and can be completed within 4 min. In 2019, Ma et al. (2019) translated the RGA scale into Chinese. The item-level content validity index (I-CVI) of the Chinese version ranges from 0.800 to 1.000, with a scale-level content validity index (S-CVI) of 0.988. The Cronbach’s α coefficients for the four subscales were 0.511, 0.758, 0.809, and 0.663, respectively.

### Other variables

A questionnaire was used to collect socio-demographic and health-related data from the participants. The variables included age, gender, education level, marital status, living arrangement, smoking history, alcohol consumption history, exercise status, body mass index (BMI), grip strength, history of falls, underlying diseases, medication type, and hospitalization in the past year.

### Variable definitions and criteria

(1) BMI group: <18.5 kg/m^2^ was underweight, 18.5–23.9 kg/m^2^ was normal weight, 24.0–27.9 kg/m^2^ was overweight, and ≥28.0 kg/m^2^ was obese.
(2) Frailty status: Participants were categorized into three groups based on their frailty score: frail (≥3 points), pre-frail (1–2 points), and non-frail (0 points). In this study, pre-frail and frail participants were combined into the frail group.
(3) Sarcopenia screening: A score of ≥4 indicated a positive screening for sarcopenia, and <4 indicated a negative screening.
(4) Nutritional assessment criteria: ≤14 points indicated increased nutritional risk, and >14 points indicated no nutritional risk.
(5) Cognitive status: Cognitive status was assessed as follows: normal (8–10 points), mild cognitive impairment (6–7 points), and dementia (0–5 points). In accordance with National Health and Aging Trends Study (NHATS) guidelines (Kasper, Freedman & Spillman, 2013), mild cognitive impairment and early dementia constitute a pathophysiological continuum, within which distinct diagnostic boundaries remain clinically indistinct. Therefore, participants with mild cognitive impairment and dementia were combined into the cognitive impairment group in this study.

### Statistical analysis

All scale survey data were entered into a database using EpiData 3.1 and analyzed with SPSS 24.0 (IBM Corp., Armonk, NY, USA). Categorical data were expressed as frequencies and proportions, and continuous data were presented as means ± standard deviations. Statistical methods included descriptive statistical analysis, χ^2^ tests, logistic regression analysis, and others, and all test results were considered statistically significant if *p* < 0.05. A logistic regression model adjusted for age, gender, marital status, education level, and the number of comorbidities was used to evaluate the odds ratios (OR) and construct 95% confidence intervals (CI) for the association between sleep and different geriatric syndromes.

## Results

### Demographic characteristics of participants

In this study, a total of 205 elderly emergency patients were evaluated, with an average age of 73.41 ± 7.10 years. The majority (43.4%) were aged between 70 and 79 years. Of the participants, 51.2% were female and 48.8% were male. Residential distribution indicated that 68.8% lived in urban areas, and 31.2% resided in rural areas. Educational attainment was distributed as follows: 13.7% had no formal schooling, 45.9% had completed primary school, 27.3% had completed junior high school, and 13.2% had a high school education or higher. Marital status showed that 91.2% were married, and 8.8% were single. BMI classification revealed that 9.8% were underweight, 75.6% had normal weight, and 14.6% were overweight. Regarding self-assessed health status, 29 participants (14.1%) rated their health as good, 139 (67.8%) rated it as average, and 37 (18.1%) rated it as poor. For self-assessed economic status, 37 participants (18.1%) considered their economic situation to be good, 153 (74.6%) considered it average, and 15 (7.3%) considered it poor. In terms of underlying diseases, 42 participants (20.5%) had no comorbidities, 102 (49.8%) had one comorbidity, 36 (17.6%) had two, 14 (6.8%) had three, and 11 (5.4%) had four or more comorbidities. Responses regarding medication use indicated that 35 participants (17.0%) were taking three or more long-term medications, 125 (61.0%) were taking one to two medications, and 45 (22.0%) were not taking any medications. Refer to Table 1 for detailed information.

**Table 1:** Demographic and geriatric syndrome data of elderly emergency patients (*n* = 205).

Project		Number of cases	Percentage (%)
Age	60–69	68	33.2
	70–79	89	43.4
	80–89	48	23.4
Sex	Man	105	51.2
	Women	100	48.8
Marital status	Alone	18	8.8
	Married	187	91.2
Educational attainment	Illiteracy	28	13.7
	Primary school	94	45.9
	Junior high school	56	27.3
	High school and above	27	13.2
Residence	Cities	141	68.8
	Rural areas	64	31.2
BMI	Underweight	20	9.8
	Normal	155	75.6
	Overweight	30	14.6
Self-assessment of economic status	Good	37	18.0
	Average	153	74.6
	Poor	15	7.4
Self-assessment of health status	Good	29	14.1
	Average	139	67.8
	Poor	37	18.1
Number of comorbidities	0	42	20.5
	1	102	49.8
	2	36	17.6
	3	14	6.8
	≥4	11	5.3
Number of types of long-term medication	0	45	22.0
	1–2	125	61.0
	≥3	35	17.0
History of hospitalization within the last six months	No	124	60.5
	Yes	81	39.5
Sleep duration	Insufficient sleep	75	36.6
	Normal sleep	107	52.2
	Excessive sleep	23	11.2
Daytime sleepiness (excluding naps)	No	100	48.8
	Slight	83	40.5
	Significant	22	10.7
Sleep quality	Satisfied	83	40.5
	Slight dissatisfaction	75	36.6
	Significant dissatisfaction	47	22.9
Use of sleeping pills	No	191	93.2
	Occasionally	11	5.4
	Long term	3	1.4
Frailty	Absence of frailty	112	54.6
	Pre-frailty	66	32.2
	Period of weakness	27	13.2
Sarcopenia	Negative	141	68.8
	Positive	64	31.2
Nutrition	No risk	79	38.5
	Increased risk	126	61.5
Cognitive function	Normal cognitive function	83	40.5
	Mild cognitive impairment	57	27.8
	Status of dementia	65	31.7

### Sleep status

Among the 205 elderly emergency patients, 75 (36.6%) had insufficient sleep duration, 107 (52.2%) had normal sleep duration, and 23 (11.2%) had excessive sleep duration in the past month. Regarding daytime sleepiness, 100 patients (48.8%) reported no daytime sleepiness, 83 (40.5%) experienced mild sleepiness, and 22 (10.7%) experienced significant sleepiness. In terms of sleep quality, 83 patients (40.5%) were satisfied, 75 (36.6%) were slightly dissatisfied, and 47 (22.9%) were significantly dissatisfied with their sleep quality over the past month. Concerning the use of sleeping pills, 191 patients (93.2%) did not use any sleeping pills, 11 (5.4%) occasionally used sleeping pills to aid sleep, and three (1.4%) required long-term use of sleeping pills to fall asleep (Table 1).

### Geriatric syndromes

This study primarily investigated four geriatric syndromes—frailty, sarcopenia, malnutrition, and cognitive impairment—in elderly emergency patients. The results show that 13.2% of the participants were classified as frail, 32.2% were pre-frail, 31.2% screened positive for sarcopenia, 61.5% had an increased nutritional risk, and 59.5% exhibited cognitive impairment. Among the participants, 40 (19.5%) did not have any of the four geriatric syndromes, 66 (32.2%) had only one syndrome, and 99 (48.3%) had two or more syndromes (Table 1).

### Univariate analysis of the association between sleep and the four geriatric syndromes

The results of this study showed that sleep duration, daytime sleepiness, and sleep quality significantly influenced the risk of frailty, sarcopenia, malnutrition, and cognitive impairment in elderly emergency patients (*p* < 0.05). Additionally, the use of sleeping pills was found to affect the risk of frailty, sarcopenia, and malnutrition significantly (*p* < 0.05). These results are shown in Table 2.

**Table 2:** Univariate analysis of the association between sleep and the four geriatric syndromes.

Project		Frailty		Sarcopenia		Nutrition		Cognitive function	
		Non-frailty	Frailty	Negative	Positive	No risk	Increased risk	No impairment	Have impairment
Sleep duration	Insufficient sleep	31	44	36	39	10	65	18	57
	Normal sleep	67	40	85	22	57	50	55	52
	Excessive sleep	14	9	20	3	12	11	10	13
	χ^2^	8.465		24.283		31.727		13.837	
	*p*	0.015*		<0.001*		<0.001*		0.001*	
Daytime sleepiness (excluding naps)	No	71	29	80	20	57	43	53	47
	Slight	37	46	50	33	22	61	25	58
	Significant	4	18	11	11	0	22	5	17
	χ^2^	25.987		12.295		33.258		13.080	
	*p*	<0.001*		0.002*		<0.001*		0.001*	
Sleep quality	Satisfied	60	23	74	9	49	34	46	37
	Slight dissatisfaction	37	38	45	30	27	48	23	52
	Significant dissatisfaction	15	32	22	25	3	44	14	33
	χ^2^	21.076		29.308		35.444		12.918	
	*p*	<0.001*		<0.001*		<0.001*		0.002*	
Use of sleeping pills	No	109	82	138	53	79	112	80	111
	Occasionally	2	9	2	9	0	11	2	9
	Long term	1	2	1	2	0	3	1	2
	χ^2^	6.903		13.949		9.998		2.469	
	*p*	0.032*^,^^f^		<0.001*^,^^f^		0.003*^,^^f^		0.325^f^	

**Notes:**

f Fisher’s Exact Test.

* Monte Carlo *p* < 0.05.

### Independent effects of sleep on the four geriatric syndromes

After adjusting for age, gender, marital status, education level, and number of comorbidities, patients with mild or significant daytime sleepiness were more likely to experience frailty compared to those without daytime sleepiness (OR = 2.509, *p* = 0.018; OR = 4.395, *p* = 0.048). Additionally, patients who reported mild or significant dissatisfaction with sleep quality had a higher likelihood of developing sarcopenia relative to those with good sleep quality (OR = 4.153, *p* = 0.006; OR = 5.955, *p* = 0.013). Patients with normal sleep patterns also exhibited a lower risk of malnutrition compared to those with insufficient sleep (OR = 0.353, *p* = 0.043), and those with slight daytime sleepiness showed an increased risk of malnutrition compared to those without sleep issues (OR = 3.414, *p* = 0.004). Furthermore, patients with mild daytime sleepiness were more prone to cognitive impairment than those without daytime sleepiness (OR = 2.564, *p* = 0.026). These results are shown in Table 3.

**Table 3:** Independent effects of sleep on the four geriatric syndromes.

	Frailty		Sarcopenia		Nutrition		Cognitive function	
	OR (95% CI)	*p*	OR (95% CI)	*p*	OR (95% CI)	*p*	OR (95% CI)	*p*
**Sleep duration**								
Insufficient sleep	1		1		1		1	
Normal sleep	1.543 [0.642–3.710]	0.333	0.845 [0.347–2.060]	0.711	0.353 [0.128–0.970]	0.043*	0.522 [0.203–1.344]	0.178
Excessive sleep	3.017 [0.837–10.882]	0.092	0.802 [0.169–3.808]	0.782	0.558 [0.149–2.094]	0.387	0.829 [0.223–3.080]	0.780
**Daytime sleep**								
No	1		1		1		1	
Slight	2.509 [1.170–5.376]	0.018*	1.547 [0.649–3.686]	0.325	3.414 [1.491–7.815]	0.004*	2.564 [1.119–5.875]	0.026*
Significant	4.395 [1.013–19.074]	0.048*	0.652 [0.160–2.653]	0.550	1227073704.615 [0.000–null]	0.999	1.732 [0.328–9.138]	0.517
**Sleep quality**								
Satisfied	1		1		1		1	
Slight dissatisfaction	2.197 [0.948–5.094]	0.067	4.153 [1.516–11.373]	0.006*	1.030 [0.430–2.467]	0.947	1.663 [0.697–3.972]	0.252
Significant dissatisfaction	3.232 [0.891–11.727]	0.074	5.955 [1.461–24.264]	0.013*	3.474 [0.625–19.315]	0.155	0.792 [0.199–3.148]	0.740
**Use of sleeping pills**								
No	1		1		1		1	
Occasionally	2.455 [0.356–16.920]	0.362	6.319 [0.880–45.374]	0.067	14642171.510 [0.000–null]	0.995	2.170 [0.237–19.862]	0.493
Long term	2.044 [0.092–45.500]	0.652	2.163 [0.112–41.792]	0.610	126913372.646 [0.000–null]	0.999	3.858 [0.180–82.584]	0.388

**Notes:**

OR, odds ratio; 95%CI, 95% confidence interval.

* Monte Carlo *p* < 0.05.

## Discussion

### Elderly emergency patients exhibit a high prevalence of geriatric syndromes, and their health needs require greater attention compared to younger patients

Although this study focused on the four specific geriatric syndromes of frailty, sarcopenia, malnutrition risk, and cognitive impairment, the results revealed that 80.5% of elderly emergency patients had at least one geriatric syndrome, and 48.3% had more than two such syndromes. This indicates an exceptionally high prevalence of geriatric syndromes among elderly emergency patients in China. Compared to other age groups, this population has a significantly higher need for emergency medical care, consistent with findings by Lin et al. (2021). Their research demonstrated that participants with any geriatric syndrome exhibited a higher prevalence of frequent hospitalizations, prolonged hospital stays, frequent outpatient visits, and polypharmacy compared to those without these syndromes. Therefore, early identification and intervention for elderly emergency patients are especially critical. Although geriatric syndromes may not be classified as specific diseases, they necessitate particular attention in clinical practice. It is thus essential to enhance the ability of emergency medical staff to assess geriatric syndromes, implement routine screening for these conditions in elderly emergency patients, and prioritize geriatric syndromes in emergency treatment protocols to improve health outcomes and clinical prognoses.

### Daytime sleepiness is an independent predictor of frailty in elderly emergency patients

Daytime sleepiness in the elderly is a complex phenomenon often associated with sleep disorders such as insomnia, sleep-disordered breathing, and circadian rhythm disturbances (Cesari, Calvani & Marzetti, 2017; Cheung et al., 2020). This condition arises from the interplay of neurobiological, physiological, and environmental factors. Disruptions in daytime sleep-wake cycles result from imbalances in neurotransmitters, particularly arousal-promoting monoamines, and dysregulation of sleep-inducing neurotransmitters (Besedovsky, Lange & Haack, 2019). Additionally, age-related changes in homeostasis and circadian sleep regulation, along with comorbidities and medications, can exacerbate daytime sleepiness.

As shown in Table 1, after adjusting for factors such as age, gender, marital status, education level, and number of comorbidities, individuals with mild daytime sleepiness were more likely to have frailty compared to those without daytime sleepiness, with an odds ratio (OR) of 2.509. Those with significant daytime sleepiness had an even higher risk, with an OR of 4.395. These findings are consistent with previous research, indicating that daytime sleepiness promotes frailty (Wu et al., 2023; Sun et al., 2023; Lu et al., 2024). Frailty results from a complex interplay of multi-system impairments distinct from normal aging processes. Compromised systems include chronic inflammation, weakened immune function, altered hormonal responses, reduced trace elements, abnormal coagulation pathways, and anemia (Fried et al., 2001).

The mechanisms by which daytime sleepiness contributes to frailty are not fully understood but may involve multiple pathways. First, chronic sleep deprivation due to disrupted sleep-wake cycles can be linked to hormonal imbalances, such as elevated cortisol levels and altered growth hormone secretion, impairing muscle mass maintenance and strength (Cesari, Calvani & Marzetti, 2017). Second, daytime sleepiness may reduce physical activity due to an increased need for rest during the day. This can lead to sarcopenia, a reduction in muscle mass and strength, which is a key factor in the development of frailty (Baniak et al., 2020; Aditi, Jaiswal & Verma, 2023). The systematic review and meta-analysis by Sun et al. (2023) supports the association between daytime sleepiness and decreased physical activity, further emphasizing the link between sleep disturbances and frailty. Inflammatory processes also play a role in connecting daytime sleepiness to frailty. Chronic sleep deprivation and disordered breathing are associated with elevated levels of inflammatory markers such as IL-6, CRP, and TNF-α, which are independently linked to frailty (Irwin, Olmstead & Carroll, 2016; Xu et al., 2022). Moreover, adipokines affected by sleep quality and duration, such as leptin and adiponectin, which play important roles in muscle metabolism, are also associated with frailty (Arai, Kamide & Hirose, 2019; Lana et al., 2017). Elevated leptin levels, commonly found in individuals with sleep disorders, can infiltrate skeletal muscle, causing muscle damage and increasing the risk of frailty (Mosavat et al., 2021).

### Daytime sleepiness and sleep duration are significant risk factors for malnutrition among elderly patients in emergency settings

Among elderly emergency patients, daytime sleepiness and sleep duration are significantly associated with malnutrition. Specifically, the findings of this study showed that mild daytime sleepiness was linked to an increased risk of malnutrition at a rate 3.414 times higher compared to no sleepiness, and individuals with insufficient sleep having a higher probability of nutritional risk compared to those with normal sleep duration. Insufficient sleep contributes to increased daytime somnolence and the disruption of circadian rhythms and homeostatic sleep processes, leading to elevated cortisol levels and subsequent muscle catabolism (Moraes et al., 2014). This disturbance can result in diminished appetite and food intake, as well as increased resting energy expenditure, potentially causing an energy deficit and ultimately leading to malnutrition (Wilcox et al., 2024). Additionally, elevated levels of inflammatory cytokines and reduced leptin further exacerbate the risk of malnutrition (Koc Okudur & Soysal, 2021; Jensen, 2015). Research has shown that elderly patients experiencing daytime sleepiness have significantly lower Mini Nutritional Assessment (MNA) scores and a higher prevalence of dysphagia (Mota et al., 2014). These observations suggest that daytime sleepiness may not only be a consequence of malnutrition, but that it is also associated with increased risk, underscoring the bidirectional relationship between these conditions.

### Daytime sleepiness is independently associated with cognitive impairment in elderly emergency patients

This study revealed that individuals who experience mild daytime sleepiness are at a higher risk of cognitive impairment compared to those who do not. This may be attributable to the significant impact of sleep deprivation on the structure of brain gray matter and higher cognitive impairment, thereby impairing memory and attention in a sleep-deprived state (Mullins et al., 2024). The association between daytime sleepiness and cognitive impairment in elderly patients presenting to emergency departments can be elucidated through multiple mechanisms. First, neurochemical alterations play a critical role. Daytime sleepiness is associated with decreased levels of neurochemical mediators such as orexin, histamine, and serotonin, which are essential for maintaining wakefulness and cognitive impairment (Kovrov et al., 2020). Reduced levels of these mediators can result in increased sleepiness and cognitive decline. Second, the accumulation of amyloid-beta protein is another key factor. Sleep facilitates the clearance of metabolic waste from the brain, including amyloid-beta protein linked to Alzheimer’s disease (Smagula et al., 2020). Disrupted sleep, especially excessive daytime sleepiness, may hinder this clearance process, leading to amyloid-beta accumulation and subsequent cognitive decline. Studies have also found that excessive daytime sleepiness is linked to an elevated risk of all-cause dementia and vascular dementia, further supporting this relationship (Cavaillès et al., 2022).

Sleep fragmentation and hypoxia are additional significant factors. Sleep disorders, such as sleep apnea, are prevalent among the elderly and can be linked to sleep fragmentation and intermittent hypoxia. These conditions are associated with cognitive decline by affecting cerebral blood flow and inducing neuroinflammation (Gabelle et al., 2013). Furthermore, both acute and chronic stress, often exacerbated by sleep disorders, can increase cortisol levels and inflammatory markers, both of which are linked to cognitive decline and an elevated risk of dementia (Saadi et al., 2021). Although glymphatic dysfunction may also serve as a potential link between sleep disturbances and neurodegeneration (Astara et al., 2024), the current study’s cross-sectional study design does not permit causal inferences. The relationship between daytime sleepiness and cognitive impairment is thus multifaceted and involves numerous biological and physiological mechanisms. Clinicians should therefore strive to recognize the importance of sleep disorders in assessing and managing geriatric syndromes in elderly emergency patients.

### Sleep quality is significantly associated with the risk of sarcopenia in elderly emergency patients

Sarcopenia refers to the age-related and progressive loss of whole-body muscle mass, strength, and physiological function (Sayer & Cruz-Jentoft, 2022). It is a significant contributor to the decline in physiological function observed in older adults, and the association between sleep and sarcopenia is multifaceted and complex. This study showed that individuals with mildly impaired sleep quality were 4.153 times more likely to develop sarcopenia compared to those with satisfactory sleep quality. Furthermore, individuals with markedly impaired sleep quality had a 5.955-fold increased risk. Poor sleep quality may serve as a key predictor of sarcopenia, consistent with findings by Aslam, Ma & Huh (2023). Although the underlying pathophysiological mechanisms are not fully understood, they may involve hormonal imbalances, increased levels of inflammatory cytokines, and disruption of circadian rhythms (Spira et al., 2012; Irwin et al., 2008). Sleep deprivation results in increased cortisol levels and reduced growth hormone secretion, both of which adversely affect muscle protein synthesis and breakdown (Dattilo et al., 2011). In addition, Morwani-Mangnani et al. (2022). examined how changes in the gut microbiome due to sleep disruption may be associated with sarcopenia, indicating that sleep disturbances modify gut microbial composition, potentially mediating the proinflammatory state associated with sarcopenia.

Human sleep patterns are regulated by circadian rhythms, particularly the sleep-wake cycle. Dysfunction of the thalamic filter within the cortical-striatal-thalamo-cortical (CSTC) circuit can impair the filtering of sensory input during normal sleep, leading to insomnia and affecting sleep quality. These findings are supported by a systematic review conducted by Pana et al. (2021), which reports that both poor sleep quality and short sleep duration are associated with reduced muscle strength and an elevated risk of sarcopenia. Ye et al. (2024) also reported a significant association between sarcopenia and poor sleep quality. Consequently, improving sleep quality may represent a crucial factor in mitigating the progression of sarcopenia.

### Limitations

This was a cross-sectional survey. As the sample is from a single hospital and based on convenience sampling, the results may not be fully representative of all elderly ED patients in China. Future research should consider conducting larger, multicenter studies to enhance the generalizability of the findings.

## Conclusions

In conclusion, when integrated with the existing body of literature, the findings of this study suggest that poor sleep is not merely a consequence of geriatric syndromes but also associated with increased risk of geriatric syndromes such as frailty, sarcopenia, malnutrition, and cognitive impairment. The pathophysiological mechanisms underlying this relationship are complex and involve neuroendocrine, inflammatory, and metabolic pathways. Future research should look for ways to improve sleep quality in elderly emergency patients in order to mitigate the risk of geriatric syndromes and optimize overall health outcomes.

## Supplemental Information

10.7717/peerj.20194/supp-1 Supplemental Information 1 Relevant data.

10.7717/peerj.20194/supp-2 Supplemental Information 2 Codebook.

10.7717/peerj.20194/supp-3 Supplemental Information 3 STROBE checklist.

## Additional Information and Declarations

### Data Availability

The following information was supplied regarding data availability:

The raw measurements are available in the Supplemental File.
