# Supplementary material for: Sleep and geriatric syndromes in elderly emergency patients in China: a cross-sectional survey
Source: PeerJ. 2025 Oct 14;13:e20194. doi: 10.7717/peerj.20194 (PMC12533537; doi:10.7717/peerj.20194)
Supplement: Supplemental Information 2 [file peerj-13-20194-s002.doc]

**Sex：**1=Man，2=Women

**Marital status：**1=Alone，2=Married

**Educational attainment：**1=Illiteracy，2=Primary school，3=Junior high school，4=High school，5=university and above

**Residence：**1=Cities，2=Rural areas

**Self-assessment of economic status：**1=good，2=In general，3=poor

**Self-assessment of health status：**1=good，2=In general，3=poor

**History of hospitalization within the last six months：**0=no，1=yes

**Daily sleep time：**1=Lack of sleep，2=Sleeping well，3=Sleeping too much

**Daytime sleepiness：**0=No daytime sleep，1=Slight sleepiness during the day，2=Significant daytime sleepiness

**Sleep quality：**0=Satisfied ，1=Slight dissatisfaction，2=Significant dissatisfaction

**Use of sleeping pills：**0=no，1=Occasionally，2=Long term
