# Supplementary material for: Sleep and geriatric syndromes in elderly emergency patients in China: a cross-sectional survey
Source: PeerJ. 2025 Oct 14;13:e20194. doi: 10.7717/peerj.20194 (PMC12533537; doi:10.7717/peerj.20194)
Supplement: Supplemental Information 3 [file peerj-13-20194-s003.docx]

STROBE Statement—checklist of items that should be included in reports of observational studies

|  | Item No. | Recommendation | Page  No. | Relevant text from manuscript |
| --- | --- | --- | --- | --- |
| **Title and abstract** | 1 | (*a*) Indicate the study’s design with a commonly used term in the title or the abstract | 1 | Sleep and geriatric syndromes in elderly emergency patients in China: a cross-sectional survey |
|  |  | (*b*) Provide in the abstract an informative and balanced summary of what was done and what was found | 1-2 | This study indicates that improving sleep may be a favorable factor for controlling geriatric syndromes. |
| Introduction | | | |  |
| Background/rationale | 2 | Explain the scientific background and rationale for the investigation being reported | 2-3 | Older patients often encounter more complex health issues with atypical symptom presentation, which leads to greater diagnostic and therapeutic difficulties compared to younger patients. Due to the decline in various organ functions and the instability of the internal environment, elderly patients are prone to recurrent acute episodes and rapid disease progression. Consequently, elderly patients admitted to the emergency department tend to have poorer prognoses, higher hospitalization rates, return visit rates, and mortality rates. |
| Objectives | 3 | State specific objectives, including any prespecified hypotheses | 3 | This study focuses directly on Chinese elderly emergency patients in order to evaluate their sleep patterns and the prevalence of geriatric syndromes within the Chinese emergency setting specifically, and to explore the relationship between sleep quality and various geriatric syndromes. We hope that this study will encourage medical staff to pay greater attention to the sleep status and geriatric syndromes of elderly emergency patients, thereby improving the quality of medical care for this vulnerable group. |
| Methods | | | |  |
| Study design | 4 | Present key elements of study design early in the paper | 4 | We recruited elderly patients from the emergency department of Yueyang Central Hospital in Hunan Province using a convenience sampling method from July to November, 2023. |
| Setting | 5 | Describe the setting, locations, and relevant dates, including periods of recruitment, exposure, follow-up, and data collection | 4 | We recruited elderly patients from the emergency department of Yueyang Central Hospital in Hunan Province using a convenience sampling method from July to November, 2023. |
| Participants | 6 | (*a*) *Cohort study*—Give the eligibility criteria, and the sources and methods of selection of participants. Describe methods of follow-up  *Case-control study*—Give the eligibility criteria, and the sources and methods of case ascertainment and control selection. Give the rationale for the choice of cases and controls  *Cross-sectional study*—Give the eligibility criteria, and the sources and methods of selection of participants | 4 | The sole inclusion criterion was: age ≥ 60 years, and the exclusion criteria were: severe physical conditions that posed an immediate threat to life and required emergency intervention. Information regarding the research procedures, objectives, related risks, and potential benefits was provided to eligible all elderly patients. |
|  |  | (*b*) *Cohort study*—For matched studies, give matching criteria and number of exposed and unexposed  *Case-control study*—For matched studies, give matching criteria and the number of controls per case |  |  |
| Variables | 7 | Clearly define all outcomes, exposures, predictors, potential confounders, and effect modifiers. Give diagnostic criteria, if applicable | 5 | Variable Definitions and Criteria  (1) BMI Group: <18.5 kg/m² was underweight, 18.5-23.9 kg/m² was normal weight, 24.0-27.9 kg/m² was overweight, and ≥28.0 kg/m² was obese.  (2) Frailty Status: Participants were categorized into three groups based on the frailty score: frail (≥3 points), pre-frail (1-2 points), and non-frail (0 points). In this study, pre-frail and frail participants were combined into the frail group.  (3) Sarcopenia Screening: A score of ≥4 indicated a positive screening for sarcopenia, and <4 indicated a negative screening.  (4) Nutritional Assessment Criteria: ≤14 points indicated increased nutritional risk, and >14 points indicated no nutritional risk.  (5) Cognitive Status: Cognitive status was assessed as follows: normal (8-10 points), mild cognitive impairment (6-7 points), and dementia (0-5 points). In this study, participants with mild cognitive impairment and dementia were combined into the cognitive impairment group. |
| Data sources/ measurement | 8* | For each variable of interest, give sources of data and details of methods of assessment (measurement). Describe comparability of assessment methods if there is more than one group | 5 | Sleep Assessment：We utilized four questions to assess the sleep status of the patients. Sleep duration was evaluated using the question "How many hours do you sleep each night?" Participants were categorized into three groups based on their self-reported sleep duration: insufficient sleep (≤ 6 hours), normal sleep (6-8 hours), and excessive sleep (≥ 9 hours). Daytime sleepiness and sleep quality were self-assessed by patients based on their experiences over the past month. If these symptoms occurred at least three times per week, participants circled the corresponding self-assessment result. The use of sleeping pills was also self-reported by patients based on their usage in the past month and was categorized into three groups: no use, occasional use, and long-term use.  Geriatric Syndrome Assessment：The Rapid Geriatric Assessment (RGA) is widely utilized to evaluate geriatric syndromes in elderly emergency patients. This scale was developed by Morley based on the Comprehensive Geriatric Assessment (CGA) and comprises four subscales: FRAIL, SARC-F, Simplified Nutritional Appetite Questionnaire (SNAQ), and Rapid Cognitive Screen (RCS), totaling 18 items. It primarily assesses core health issues in elderly patients and can be completed within 4 minutes. In 2019, Ma Lin translated the RGA scale into Chinese. The item-level content validity index (I-CVI) of the Chinese version ranges from 0.800 to 1.000, with a scale-level content validity index (S-CVI) of 0.988. The Cronbach's α coefficients for the four subscales are 0.511, 0.758, 0.809, and 0.663, respectively. |
| Bias | 9 | Describe any efforts to address potential sources of bias |  |  |
| Study size | 10 | Explain how the study size was arrived at | 4 | According to Kendall's method for rough sample size estimation, the sample size should be at least 5 to 10 times the number of variables. In this study, with 15 variables, the recommended sample size would range from 75 to 150. A total of 205 elderly patients were ultimately included. |

Continued on next page

| Quantitative variables | 11 | Explain how quantitative variables were handled in the analyses. If applicable, describe which groupings were chosen and why | 5 | Variable Definitions and Criteria  (1) BMI Group: <18.5 kg/m² was underweight, 18.5-23.9 kg/m² was normal weight, 24.0-27.9 kg/m² was overweight, and ≥28.0 kg/m² was obese.  (2) Frailty Status: Participants were categorized into three groups based on the frailty score: frail (≥3 points), pre-frail (1-2 points), and non-frail (0 points). In this study, pre-frail and frail participants were combined into the frail group.  (3) Sarcopenia Screening: A score of ≥4 indicated a positive screening for sarcopenia, and <4 indicated a negative screening.  (4) Nutritional Assessment Criteria: ≤14 points indicated increased nutritional risk, and >14 points indicated no nutritional risk.  (5) Cognitive Status: Cognitive status was assessed as follows: normal (8-10 points), mild cognitive impairment (6-7 points), and dementia (0-5 points). In this study, participants with mild cognitive impairment and dementia were combined into the cognitive impairment group. |
| --- | --- | --- | --- | --- |
| Statistical methods | 12 | (*a*) Describe all statistical methods, including those used to control for confounding | 5 | All scale survey data were entered into a database using EpiData 3.1 and analyzed statistically with SPSS 24.0. Categorical data were expressed as frequencies and proportions, and continuous data were presented as means ± standard deviations. Statistical methods included descriptive statistical analysis, χ² tests, logistic regression analysis, and others, and all test results were considered statistically significant if P < 0.05. A logistic regression model adjusted for age, gender, marital status, education level, and the number of comorbidities was used to evaluate the odds ratios (OR) and construct 95% confidence intervals (CI) for the association between sleep and different geriatric syndromes. |
|  |  | (*b*) Describe any methods used to examine subgroups and interactions |  |  |
|  |  | (*c*) Explain how missing data were addressed |  |  |
|  |  | (*d*) *Cohort study*—If applicable, explain how loss to follow-up was addressed  *Case-control study*—If applicable, explain how matching of cases and controls was addressed  *Cross-sectional study*—If applicable, describe analytical methods taking account of sampling strategy |  |  |
|  |  | (*e*) Describe any sensitivity analyses |  |  |
| Results | | | | |
| Participants | 13* | (a) Report numbers of individuals at each stage of study—eg numbers potentially eligible, examined for eligibility, confirmed eligible, included in the study, completing follow-up, and analysed | 6 | In our study, a total of 205 elderly emergency patients were evaluated |
|  |  | (b) Give reasons for non-participation at each stage |  |  |
|  |  | (c) Consider use of a flow diagram |  |  |
| Descriptive data | 14* | (a) Give characteristics of study participants (eg demographic, clinical, social) and information on exposures and potential confounders | 6 | In our study, a total of 205 elderly emergency patients were evaluated, with an average age of 73.41 ± 7.10 years. The majority (43.4%) were aged between 70 and 79 years. Of the participants, 51.2% were female and 48.8% were male. Residential distribution indicated that 68.8% lived in urban areas, and 31.2% resided in rural areas. Educational attainment was distributed as follows: 13.7% had no formal schooling, 45.9% had completed primary school, 27.3% had completed junior high school, and 13.2% had a high school education or higher. Marital status showed that 91.2% were married, and 8.8% were single. BMI classification revealed that 9.8% were underweight, 75.6% had normal weight, and 14.6% were overweight. Regarding self-assessed health status, 29 participants (14.1%) rated their health as good, 139 (67.8%) rated it as average, and 37 (18.1%) rated it as poor. For self-assessed economic status, 37 participants (18.1%) considered their economic situation to be good, 153 (74.6%) considered it average, and 15 (7.3%) considered it poor. In terms of underlying diseases, 42 participants (20.5%) had no comorbidities, 102 (49.8%) had one comorbidity, 36 (17.6%) had two, 14 (6.8%) had three, and 11 (5.4%) had four or more comorbidities. Medication use responses indicated that 35 participants (17.0%) were taking three or more long-term medications, 125 (61.0%) were taking one to two medications, and 45 (22.0%) were not taking any medications. Refer to Table 1 for detailed information. |
|  |  | (b) Indicate number of participants with missing data for each variable of interest |  |  |
|  |  | (c) *Cohort study*—Summarise follow-up time (eg, average and total amount) |  |  |
| Outcome data | 15* | *Cohort study*—Report numbers of outcome events or summary measures over time |  |  |
|  |  | *Case-control study—*Report numbers in each exposure category, or summary measures of exposure |  |  |
|  |  | *Cross-sectional study—*Report numbers of outcome events or summary measures | 7 | After adjusting for age, gender, marital status, education level, and number of comorbidities, patients with mild or significant daytime sleepiness were more likely to experience frailty compared to those without daytime sleepiness (OR=2.509, p=0.018; OR=4.395, p=0.048). Additionally, patients who reported mild or significant dissatisfaction with sleep quality had a higher likelihood of developing sarcopenia relative to those with good sleep quality (OR=4.153, p=0.006; OR=5.955, p=0.013). Patients with normal sleep patterns also exhibited a lower risk of malnutrition compared to those with insufficient sleep (OR=0.353, p=0.043), and those with slight daytime sleepiness showed an increased risk of malnutrition compared to those without sleep issues (OR=3.414, p=0.004). Furthermore, patients with mild daytime sleepiness were more prone to cognitive impairment than those without daytime sleepiness (OR=2.564, p=0.026). |
| Main results | 16 | (*a*) Give unadjusted estimates and, if applicable, confounder-adjusted estimates and their precision (eg, 95% confidence interval). Make clear which confounders were adjusted for and why they were included | 7 | After adjusting for age, gender, marital status, education level, and number of comorbidities, patients with mild or significant daytime sleepiness were more likely to experience frailty compared to those without daytime sleepiness (OR=2.509, p=0.018; OR=4.395, p=0.048). Additionally, patients who reported mild or significant dissatisfaction with sleep quality had a higher likelihood of developing sarcopenia relative to those with good sleep quality (OR=4.153, p=0.006; OR=5.955, p=0.013). Patients with normal sleep patterns also exhibited a lower risk of malnutrition compared to those with insufficient sleep (OR=0.353, p=0.043), and those with slight daytime sleepiness showed an increased risk of malnutrition compared to those without sleep issues (OR=3.414, p=0.004). Furthermore, patients with mild daytime sleepiness were more prone to cognitive impairment than those without daytime sleepiness (OR=2.564, p=0.026). |
|  |  | (*b*) Report category boundaries when continuous variables were categorized |  |  |
|  |  | (*c*) If relevant, consider translating estimates of relative risk into absolute risk for a meaningful time period |  |  |

Continued on next page

| Other analyses | 17 | Report other analyses done—eg analyses of subgroups and interactions, and sensitivity analyses |  |  |
| --- | --- | --- | --- | --- |
| Discussion | | | | |
| Key results | 18 | Summarise key results with reference to study objectives | 11 | In conclusion, when integrated with the existing body of literature, our findings suggest that poor sleep is not merely a consequence of geriatric syndromes but also serves as a contributing factor. The pathophysiological mechanisms underlying this relationship are complex and involve neuroendocrine, inflammatory, and metabolic pathways. Future research should look for ways to improve sleep quality in elderly emergency patients in order to mitigate the risk of geriatric syndromes and optimize overall health outcomes. |
| Limitations | 19 | Discuss limitations of the study, taking into account sources of potential bias or imprecision. Discuss both direction and magnitude of any potential bias | 11 | This was a cross-sectional survey. Given the unique characteristics of elderly emergency patients, the sample was limited to a single hospital, resulting in a relatively small and potentially non-representative sample size. Future research should consider conducting larger, multicenter studies to enhance the generalizability of the findings. |
| Interpretation | 20 | Give a cautious overall interpretation of results considering objectives, limitations, multiplicity of analyses, results from similar studies, and other relevant evidence | 7-11 |  |
| Generalisability | 21 | Discuss the generalisability (external validity) of the study results | 7-11 |  |
| Other information | |  | | |
| Funding | 22 | Give the source of funding and the role of the funders for the present study and, if applicable, for the original study on which the present article is based | 12 | This work was founded by Hunan Provincial Health and Wellness Committee (No: D202314019348), Yueyang Science and Technology Department in China (No: 2024-11), Project of Yueyang Central Hospital (No:YYSZXYN2023011). |

*Give information separately for cases and controls in case-control studies and, if applicable, for exposed and unexposed groups in cohort and cross-sectional studies.

**Note:** An Explanation and Elaboration article discusses each checklist item and gives methodological background and published examples of transparent reporting. The STROBE checklist is best used in conjunction with this article (freely available on the Web sites of PLoS Medicine at http://www.plosmedicine.org/, Annals of Internal Medicine at http://www.annals.org/, and Epidemiology at http://www.epidem.com/). Information on the STROBE Initiative is available at www.strobe-statement.org.
